# Fungal mycobiome-mediated immune response: a non-negligible promoter in pancreatic oncogenesis and chemoresistance

**DOI:** 10.20517/cdr.2023.06

**Published:** 2023-05-10

**Authors:** Yaling Jiang, Valentina Donati, Godefridus J. Peters, Elisa Giovannetti, Dong Mei Deng

**Affiliations:** ^1^Department of Preventive Dentistry, Academic Center for Dentistry Amsterdam (ACTA), University of Amsterdam and Vrije Universiteit Amsterdam, Amsterdam 1081 LA, Netherlands.; ^2^Department of Medical Oncology, Amsterdam University Medical Centers, location VUMC, Vrije Universiteit Amsterdam, Amsterdam 1081 HV, Netherlands.; ^3^Unit of Pathological Anatomy 2, Azienda Ospedaliero-Universitaria Pisana, Pisa 56100, Italy.; ^4^Department of Biochemistry, Medical University of Gdansk, Gdansk 80-210, Poland.; ^5^Fondazione Pisana per la Scienza ONLUS, San Giuliano Terme, Pisa 56017, Italy.

**Keywords:** Mycobiome, pancreatic cancer, chemoresistance, interleukin 33, type 2 immune cells

## Abstract

Pancreatic ductal adenocarcinoma (PDAC) is one of the most lethal cancers in humans due to late diagnosis and poor response to treatments. The tumor microenvironment (TME) of PDAC is characterized by a distinctive, suppressive immune profile, which inhibits the protective functions of anti-tumor immunity and thereby contributes to PDAC progression. Recently, the study of Alam *et al.* discovered for the first time that the intratumoral fungal mycobiome could contribute to the recruitment and activation of type 2 immune cells in the TME of PDAC via enhancing the secretion of a chemoattractant, interleukin (IL-) 33. In this article, we reviewed the important findings of this study. Together with our findings, we synthetically discussed the role of the fungal mycobiome in orchestrating the immune response and thereby modulating tumor progression.

## MAIN TEXT

The recent development of various –omics approaches has greatly expanded our knowledge of the diverse fungal species colonizing different body sites, which constitute an important component of the human microbiome, termed the “mycobiome”^[1,2]^. Traditionally, members of the human mycobiome, such as the well-known *Candida* spp., are known as opportunistic pathogens which reside in most healthy individuals and cause local or systemic infectious diseases only under certain circumstances. However, a growing body of evidence has suggested that the mycobiome plays a critical role in the onset and progression of cancers^[3]^. A noteworthy example is the implication of the mycobiome in the carcinogenesis of pancreatic ductal adenocarcinoma (PDAC).

PDAC is a highly aggressive malignancy with a 5-year overall survival (OS) rate of around 10%, ranking the fourth leading cause of cancer-related deaths in the Western world^[4]^. The dismal prognosis mainly results from a lack of specific symptoms for early diagnosis, the early metastatic spread and the poor response to available treatments^[5]^. Previously, a seminal study discovered that PDAC harbors a distinct mycobiome profile as compared to that of the normal pancreas, while fungal ablation with antifungal treatments showed protective effects against oncogenic progression^[6]^. These results suggested the potential of the mycobiome in PDAC as a new target for the development of novel biomarkers and therapeutic strategies^[7]^.

The tumor microenvironment (TME) of PDAC is characterized by a distinctive immune profile dominated by immune-suppressive cells including T_H_2 cells and innate lymphoid cells 2 (ILC2), which can inhibit the functions of anti-tumor T cell immunity and thereby contribute to PDAC progression^[8]^. In addition, these infiltrated T_H_2 cells have also been found to fuel PDAC progression in the early stage of tumorigenesis via the secreted type 2 pro-tumorigenic cytokines, such as interleukin (IL-) 4 and IL-13^[9]^.

**Figure 1:**
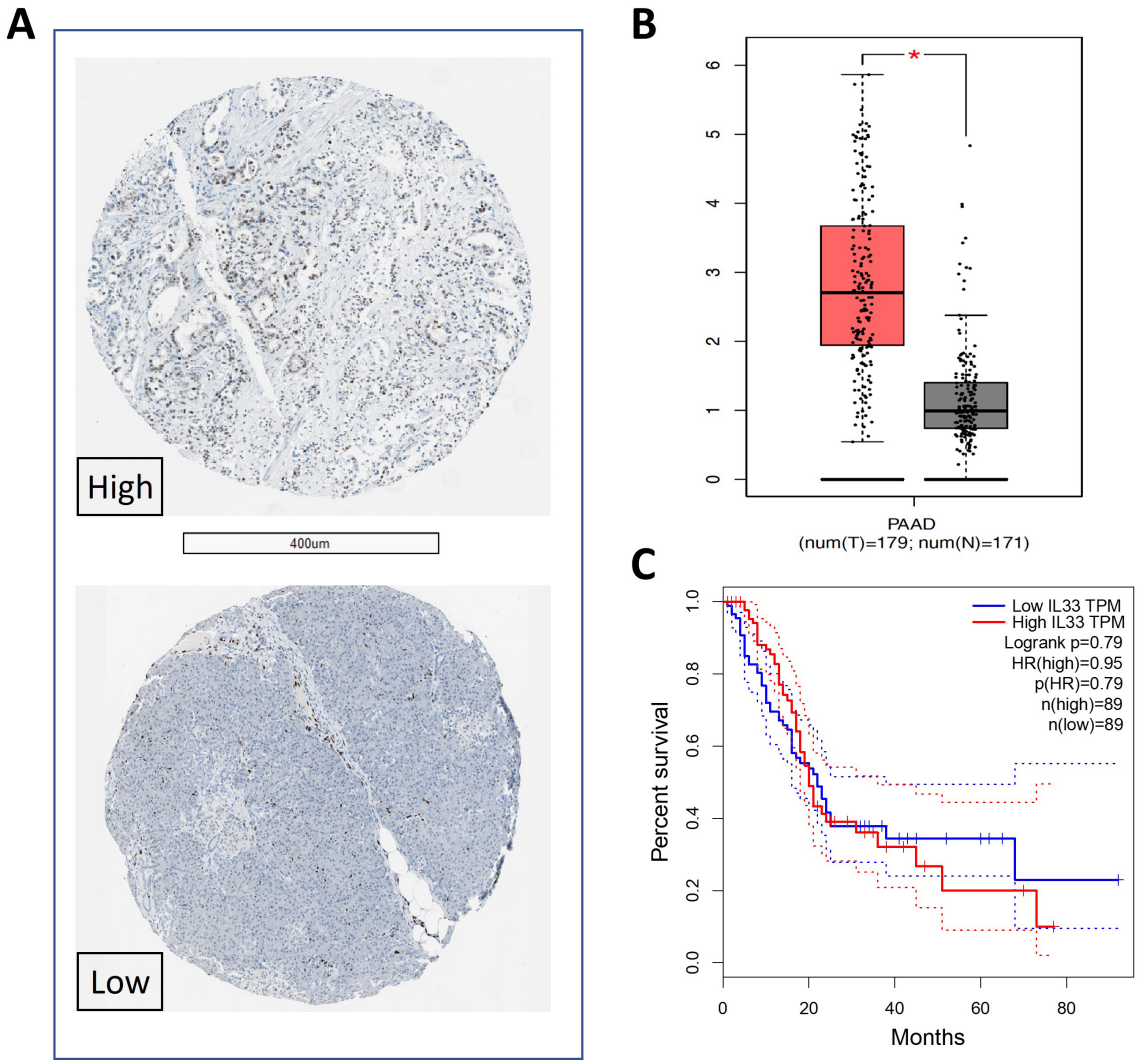
IL-33 expression and outcome in PDAC. (A) Representative images showing differential expression (high *vs.* low) of IL-33 in cores of tissue microarrays including specimens from two PDAC patients^[24]^. The immunohistochemical staining was performed using the IL-33 antibody AF3626 (R&D systems), as reported by Alam *et al*^[10]^; (B) the mRNA expression of IL-33 in PDAC was evaluated using the web-based genomics analysis and visualization platform GEPIA, analyzing the RNA sequencing expression data of 9,736 tumors and 8,587 normal samples from the TCGA and the GTEx projects, including 179 PDAC and 117 normal pancreatic tissues; (C) the levels of IL-33 mRNA expression did not correlate with overall survival in the TCGA-PAAD database.

Most recently, Alam and collaborators^[10]^ revealed for the first time that the intratumoral mycobiome could enhance the secretion of the chemoattracting cytokine IL-33 from cancer cells, which subsequently recruited and activated T_H_2 and ILC2 cells in the TME of PDAC, thus promoting pancreatic oncogenesis. This study was the first to show that TME of PDAC has an increased infiltration of T_H_2 and ILC2 cells as compared to the normal pancreas both in a PDAC mouse model and in human PDAC samples^[10]^. In order to determine the chemotactic factors secreted by cancer cells that may recruit and activate these immune cells, the authors also conducted a transcriptomic analysis of multiple PDAC cell lines and identified a 30-fold upregulation of IL-33, which was mediated by oncogenic *Kras*^G12D^ signaling. Immunohistochemistry staining showed that IL-33 expression was indeed relatively high in human PDAC tissues, while it was undetectable or below 25% nuclear staining in exocrine cells in normal pancreas specimens in the Human Protein Atlas database (https://www.proteinatlas.org, Figure 1A). IL-33 is known as a potent activator of T_H_2 and ILC2 cells^[11]^. In order to elucidate the requirement of IL-33 expression by cancer cells to recruit type 2 immunocytes, the authors depleted IL-33 in cancer cells by lentivirus transduction of small hairpin RNA in a syngeneic orthotopic model of PDAC. The IL-33 depletion reduced both T_H_2 and ILC2 infiltration in the TME and functionally inactivated the resident ILC2 cells that were already present within the TME. The decreased IL-33 expression in cancer cells also resulted in reduced tumor burden and increased survival. Taken together, these results indicated that cancer-cell-derived IL-33 recruits and activates type 2 immune cells into the TME of PDAC.

Other experiments focused on the role of the mycobiome in PDAC on IL-33 secretion as well as in PDAC tumorigenesis^[10]^. Using both 18S internal transcribed spacer (ITS) sequencing and fluorescence *in situ* hybridization, a higher load of fungi was found in the PDAC specimens as compared to the normal pancreas, with *Malassezia* being the most abundant genus, in accordance with previous findings^[6]^. In addition, in both studies, the fungal depletion or repopulation was shown to retard or accelerate PDAC tumor growth, respectively. However, various mechanisms may be responsible. *M. globosa* was shown to promote tumor progress^[6]^ via mannose-binding lectin that can recognize fungal pathogens and the subsequent activation of C3 complement cascade which belongs to innate immunity^[12]^. Interestingly, *M. globosa* was also shown to be involved in regulating the adaptive immune response to promote tumor growth by facilitating the extracellular expression of IL-33 and consequently enhancing the infiltration of T_H_2 and ILC2 cells^[10]^.

However, IL-33 is a member of the IL-1 cytokine family^[13]^ and it is well known for its dichotomous functions, acting both as a traditional extracellular cytokine and as a nuclear transcription factor^[14]^. Unlike the traditional inducible cytokines, IL-33 is constitutively expressed by several cells including human endothelial and epithelial cells. The full-length IL-33 can translocate to the nucleus upon synthesis and be stored there^[15,16]^. This nuclear IL-33 might function as a transcriptional repressor to decrease inflammation^[17]^. Once released or secreted, the extracellular IL-33 can be cleaved to its more active form and induce the type 2 immune response^[11,18]^. Interestingly, there are different opinions on the function of type 2 immune response in intestinal immunity and in the development of pancreatitis and PDAC. Some studies showed that IL-33 deficient mice were highly susceptible to colitis, colorectal cancer and pancreatitis^[19,20]^, which suggest a protective function of IL-33. However, other studies showed increased IL-33 levels in biopsies obtained from patients with active inflammatory bowel disease^[21]^, while elevated serum IL-33 was found in patients with severe acute pancreatitis^[22]^. Opposite findings described that the IL-33-induced ILC2 infiltration in PDAC cells correlated positively with long-time survival in patients^[23]^. In The Cancer Genome Atlas (TCGA) database, the mRNA expression of IL-33 is significantly higher in PDAC tissues compared to normal pancreatic tissues but was not associated with a significantly different OS [Figure 1B and 1C].

However, in many human cancers including PDAC, it was found that the fragile X mental retardation protein (FMRP) repressed immune attack by up-regulating IL-33 together with tumor-secreted protein S and extracellular vesicles (EVs), which promote M2-like tumor-associated macrophages, while down-regulating the chemoattractant C-C motif chemokine ligand 7^[25]^. Of note, FMRP mRNA and protein expression levels were not associated with clinical outcomes in several cohorts of cancer patients, but a gene signature reflecting FMRP’s cancer regulatory activity (with 156 genes, including IL-33) was prognostic for reduced OS across multiple human cancers. These discrepancies underline the importance of understanding the role of IL-33, its induced type 2 immune response and the network of genes and cells in the TME that contribute to the capability of PDAC to evade immune destruction and resist chemotherapy. Elucidation of the processes is essential before designing novel treatment strategies.

Since the mycobiome is living in symbiosis with bacteria as commensals in the human body, it is very likely that the mycobiome exerts an important influence on the microbiome. The involvement of microbiome in the development of PDAC and its chemoresistance has been convincingly demonstrated by several studies^[26-29]^. Bacterial taxa, Proteobacteria (*Pseudoxanthomonas*) and Actinobacteria (*Saccharopolyspora* and *Streptomyces*), are positively correlated to the short-term survival of PDAC patients^[29]^. The 18S ITS sequencing can only detect the presence of fungal mycobiome but not the presence of microbiome. It is therefore unclear whether microbiome could function similarly to the mycobiome, inducing the secretion of extracellular IL-33 and thus activating the type 2 immune response. The factors which can trigger the release or secretion of extracellular IL-33 are not fully identified. Besides fungi or fungal components^[10]^, it has been shown that cellular injury or death is one of the mechanisms by which IL-33 reaches the extracellular environment^[30]^. Hence IL-33 will also act as an alarm when there is a breach in the primary defenses of intestinal epithelium against pathogens and other threats^[18]^. Other factors, such as extracellular ATP concentrations, mechanical stress or oxidative stress, can also enhance the secretion of IL-33. Extracellular ATP concentrations are regulated by ectonucleotidases CD39 and CD73, which are known to play a role in immune function as well^[31,32]^. Multiple bacterial species, such as *Klebsiella pneumoniae* (for autoimmune pancreatitis)^[33]^, *Helicobacter pylori* (for gastric ulcers)^[34]^, *Staphylococcus aureus* (for lung infection)^[35]^, have been shown to enhance the secretion of IL-33, indicating the involvement of tumoral microbiome, possibly together with mycobiome, in orchestrating the innate and adaptive immunity and modulate tumor progression.

Furthermore, mycobiome may also play a role in chemoresistance. Aykut *et al.*^[6]^ have shown that fungal ablation via antifungal treatment enhanced the efficacy of gemcitabine-based chemotherapy in PDAC-bearing mice. In humans, it was suggested that the gut mycobiome might modulate the response to preoperative chemotherapy (gemcitabine-cisplatin) in patients with bladder cancer^[36]^. Compared to the non-responders, the responders had a distinct mycobiome featured by a higher diversity and lower abundance of *Agaricomycetes* and *Sacchaaromycetes*. However, the mechanisms underlying mycobiome-induced chemoresistance are still unclear. There are several hypotheses: (1) mycobiome may confer chemoresistance through metabolism and enzymatic degradation of chemotherapeutic drugs, similar to the previously reported intratumoral bacteria-mediated chemoresistance^[27]^; (2) studies on breast cancer have identified IL-33 as a key driver of chemoresistance of tumor cells. IL-33 overexpression transformed tumor cells into polyploidal giant cancer cells that are highly resistant to chemotherapy due to their dormancy or abnormal cell cycle^[37,38]^. Thus, the fungal mycobiome may also elicit chemoresistance of cancer cells via the enhanced secretion of IL-33, as shown in the current evaluated study of Alam *et al.*^[10]^.

**Figure 2:**
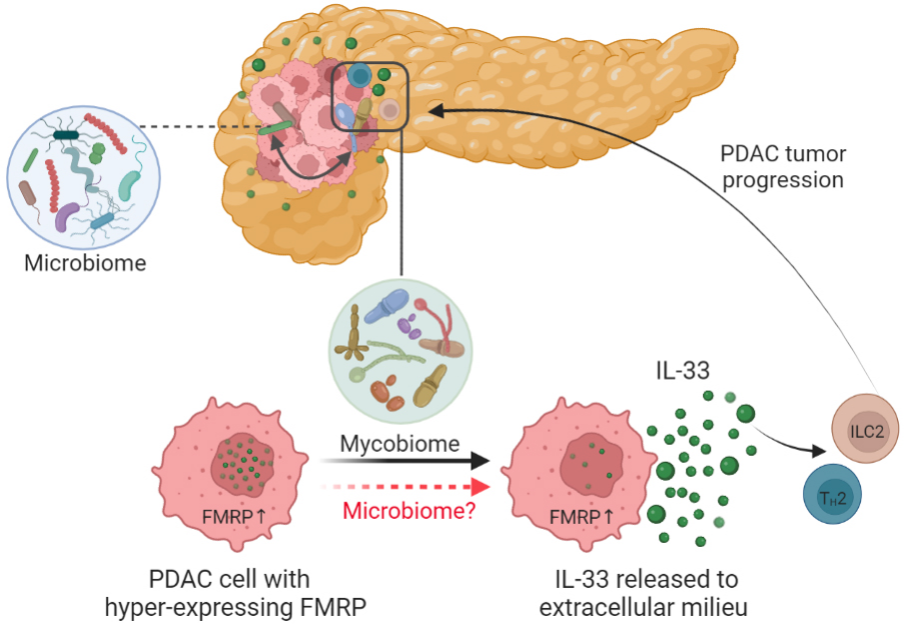
Intratumoral mycobiome can facilitate the extracellular secretion of interleukin (IL-) 33 from the nuclear of pancreatic ductal adenocarcinoma (PDAC) cells, which recruit the pro-tumorigenic immune cells such as T_H_2 and ILC2 cells to the tumor microenvironment and thus accelerate PDAC tumor progression. Notably, bacteria species have been shown to be able to enhance the secretion of IL-33, and possibly together with mycobiome, intratumoral microbiome may also exert a role in orchestrating the host immune response and modulating tumor progression. FMRP, fragile X mental retardation protein. (Created with BioRender.com).

In summary, recent data revealed that the intratumoral fungal mycobiome can contribute to PDAC pathogenesis by stimulating the extracellular secretion of IL-33 from cancer cells, thus driving the recruitment and activation of T_H_2 and ILC2 cells in TME of PDAC and promoting tumor progression [Figure 2]. Recent studies suggest that additional mechanisms, including modulation of FMRP and interaction with other fungi and bacteria, play a pivotal role in the impact of IL-33 as “friend or foe” in PDAC [Figure 2]. These findings may provide new insights for the development of novel therapeutic strategies for overcoming PDAC chemoresistance by targeting the intratumoral mycobiome and correlated factors. Nevertheless, research on fungal mycobiome in PDAC is still at the infant stage. More studies are needed to illustrate how fungal mycobiome and its interaction with intratumoral bacteria can influence the oncogenesis and chemoresistance of PDAC. Furthermore, clinical studies should also be conducted to understand the prevalence of fungal infection in PDACs, the heterogeneity of IL-33 expression and the tumor-stage associated IL-33 expression, which may assist in the discovery of novel biomarkers for monitoring disease progression.

## DECLARATIONS

### Availability of data and materials

Data supporting the findings of Figure 1 are available from the authors upon request.

### Copyright

© The Author(s) 2023.
